# Research on Bounded Rationality of Fuzzy Choice Functions

**DOI:** 10.1155/2014/928279

**Published:** 2014-03-23

**Authors:** Xinlin Wu, Yong Zhao

**Affiliations:** ^1^Department of Systems Science and Engineering, Huazhong University of Science and Technology, Wuhan, Hubei 430074, China; ^2^Department of Mathematics, Hubei University of Education, Wuhan, Hubei 430205, China

## Abstract

The rationality of a fuzzy choice function is a hot research topic in the study of fuzzy choice functions. In this paper, two common fuzzy sets are studied and analyzed in the framework of the Banerjee choice function. The complete rationality and bounded rationality of fuzzy choice functions are defined based on the two fuzzy sets. An assumption is presented to study the fuzzy choice function, and especially the fuzzy choice function with bounded rationality is studied combined with some rationality conditions. Results show that the fuzzy choice function with bounded rationality also satisfies some important rationality conditions, but not vice versa. The research gives supplements to the investigation in the framework of the Banerjee choice function.

## 1. Introduction

The rationality of a choice function is an important issue in the research of classical decision theory. The rationality is expressed by the existence of a preference relation with respect to which the decision behavior fulfills a condition of optimum. For a choice function the problem is whether or not it is rational. The rationality has been widely studied in the context of preference modeling and choice functions. To deal with this issue, various rationality conditions have been presented by a number of authors. Among these conditions, the most popular are the Weak Axiom of Revealed Preference (WARP) by Samuelson [1], the Strong Axiom of Revealed Preference (SARP) by Houthakker [2], the rationality conditions C1–C5 by Arrow [3], the Strong Congruence Axiom (SCA) by Richter [4], and the Weak Congruence Axiom (WCA) and some contraction-expansion conditions by Sen [5]. Moreover, Arrow and Sen investigated the relationships among these rationality conditions and obtained some important results. These results laid the foundation for the rationalization of choice functions.

Along with the development of decision sciences, it was often difficult for people to obtain accurate data because the complexity of the decision making system also increased. Moreover, the participation of human is indispensable to the decision making process. The preferences between alternatives are described by crisp preference relations, which have certain limitations. The fuzzy preferences were modelled in the study of choice functions by Orlovsky in 1978. He argued that sometimes it was difficult for a decision maker to state definitely the preferences between alternatives. Therefore, the degree of preference between alternatives can be reflected by a number in [0, 1]. Based on fuzzy preferences, Orlovsky proposed a choice function in fuzzy environment, which is named as Orlovsky choice function [6]. From then on, many studies have been dedicated to this subject. Roubens [7] extended the Orlovsky choice function and discussed several nonfuzzy choice functions based on fuzzy preferences. The rationality properties of those choice functions were given. Banerjee [8] further argued that “If the preferences are permitted to be fuzzy, it seems natural to permit the choice function to be fuzzy as well. This also tallies with experience. Moreover, this fuzziness of choice is, at least potentially, observable.” Based on this argument, Banerjee fuzzified the range of the choice function and gave the general definition of fuzzy choice function, that is, Banerjee choice function. Moreover, three fuzzy congruence conditions were proposed to describe the rationality of the fuzzy choice function and it was verified that these conditions were necessary and sufficient conditions for the rationalization of the fuzzy choice function. Georgescu [9] extended Banerjee's work and gave a new definition of fuzzy choice function by fuzzifying the available domain. This is the most general definition of a fuzzy choice function in the literature, which is called Georgescu choice function. Georgescu [10] further fuzzified some rationality conditions which were proposed by Richter, Arrow, and Sen. Although the Georgescu choice function is more general, many of her conclusions were obtained by some special conditions (such as intuitionistic negation and the special* t*-norms).

Similar to the crisp case, the study of fuzzy choice function mainly focused on the study of rationality conditions of fuzzy choice functions and their relationships. Wang [11] further studied Banerjee's three fuzzy congruence conditions and proved that they are not independent. Georgescu [12] proposed acyclic rationality indicators in the study of rationality of a fuzzy choice function and studied these indicators. Wu et al. [13] fuzzified some important rationality conditions of fuzzy choice functions in the framework of the Banerjee choice function and obtained some results under the assumption that every involved choice set is normal. This paper is mainly inspired by Wu et al. [13]; the differences between them can be summed up as follows. (1) In [13], the main conclusions are obtained under the assumption that every involved choice set is normal. However I do not have the assumption in this paper. (2) In [13], the main conclusions are obtained based on the condition “*C*(*S*) = *G*(*S*, *R*)” while the main conclusions in this paper are obtained based on the condition “*C*(*S*) = *M*(*S*, *R*).” (3) In [13], the rationality properties of fuzzy choice functions are studied based on the fuzzy revealed preference, while the main conclusions are obtained based on the fuzzy preference in this paper. Obviously, the fuzzy revealed preference is a stronger condition.

In this paper, two common fuzzy sets (*G*(*S*, *R*) and *M*(*S*, *R*)) are studied in the framework of the Banerjee choice function, the concepts about complete rationality and bounded rationality of the fuzzy choice function are presented, and the bounded rationality of fuzzy choice function is studied. The paper is organized as follows.  Section 2 gives some concepts including* t*-norms, fuzzy preference, and fuzzy choice function.  Section 3 studies the conditions for the nonemptiness of two fuzzy sets, *G*(*S*, *R*) and *M*(*S*, *R*).  Section 4 presents an assumption about a fuzzy choice function and gives the concepts about complete rationality and bounded rationality of the fuzzy choice function, and especially the bounded rationality of the fuzzy choice function combined with a certain condition is studied.  Section 5 summarizes this paper.

## 2. Preliminaries

In this section, we recall some used basic notions and notations in this paper.

### 2.1. *t-*Norm

**Definition 1**

A mapping *T* : [0,1] × [0,1] → [0,1] is called a* t-*norm if it satisfies the following.

1. Symmetry: ∀*x*, *y* ∈ [0,1], *T*(*x*, *y*) = *T*(*y*, *x*).
2. Monotonicity: ∀*x*_1_ ≤ *x*_2_, *y*_1_ ≤ *y*_2_, *T*(*x*_1_, *y*_1_) ≤ *T*(*x*_2_, *y*_2_).
3. Associativity: ∀*x*, *y*, *z* ∈ [0,1], *T*(*T*(*x*, *y*), *z*) = *T*(*x*, *T*(*y*, *z*)).
4. Boundary condition: ∀*x* ∈ [0,1],  *T*(1, *x*) = *x*.

Here, *T*(*T*(*x*, *y*), *z*) or *T*(*x*, *T*(*y*, *z*)) is denoted as *T*(*x*, *y*, *z*) in this paper. A* t-*norm *T* is called continuous if it is continuous as a function on the unit interval. The three most important* t-*norms are the minimum, *T*(*x*, *y*) = min⁡(*x*, *y*), the Lukasiewicz* t-*norm, *T*(*x*, *y*) = max⁡(*x* + *y* − 1,0), and the (algebraic) product, *T*(*x*, *y*) = *xy*.

### 2.2. Fuzzy Choice Functions and Fuzzy Preference Relations

Let *X* denote the nonempty finite set of alternatives. Let *H* denote the set of all nonempty, finite, and crisp subsets of *X*. *F* denotes the set of all nonempty fuzzy subsets of *X* with finite supports.

**Definition 2 (see [8])**

A fuzzy choice function is a function *C* : *H* → *F* such that ∀*S* ∈ *H*,  Supp  *C*(*S*)⊆*S*, where Supp  *C*(*S*) denotes the support of *C*(*S*).

**Remark 3**

For all *S* ∈ *H*,  *x* ∈ *S*, *C*(*S*) is a fuzzy set, and *C*(*S*)(*x*) represents the extent to which *x* belongs to the fuzzy set *C*(*S*) or the extent to which *x* is chosen in the set *S*. For example, let *S* = {*x*, *y*, *z*}; if *C*(*S*)(*x*) = 0.8, then it implies that the extent to which *x* is chosen in the set *S* is 0.8.

**Definition 4 (see [8])**

A fuzzy preference relation on *X* is a function *R* : *X* × *X* → [0,1].

Here, for all distinct *x*, *y* ∈ *X*, *R*(*x*, *y*) represents the degree to which *x* is preferred to *y*. Moreover, the fuzzy relation *R* on *X* is reflexive if *R*(*x*, *x*) = 1 for any *x* ∈ *X*; *R* is complete if *R*(*x*, *y*) + *R*(*y*, *x*) ≥ 1 for any *x*, *y* ∈ *X*; *R* is strongly complete if *R*(*x*, *y*) = 1 or *R*(*y*, *x*) = 1 for any *x*, *y* ∈ *X*; *R* is* T*-transitive if *T*(*R*(*x*, *y*), *R*(*y*, *z*)) ≤ *R*(*x*, *z*) for any *x*, *y*, *z* ∈ *X*; *R* is acyclic if *R*(*x*_1_, *x*_2_) > *R*(*x*_2_, *x*_1_), *R*(*x*_2_, *x*_3_) > *R*(*x*_3_, *x*_2_),…, *R*(*x*_*n*−1_, *x*_*n*_) > *R*(*x*_*n*_, *x*_*n*−1_); then *R*(*x*_1_, *x*_*n*_) ≥ *R*(*x*_*n*_, *x*_1_) for *n* ≥ 2 and any *x*_1_, *x*_2_,…, *x*_*n*_ ∈ *X*.

Like in the crisp case, a fuzzy choice function can induce various fuzzy preferences, among which the fuzzy revealed preference is the most important one.

**Definition 5 (see [8])**

Let *C* be a fuzzy choice function. The fuzzy revealed preference relation *R* on *X* is defined by ∀*x*, *y* ∈ *X*, *R*(*x*, *y*) = max⁡_{*S*∣*x*,*y*∈*S*}_ *C*(*S*)(*x*).

From Definition 5, ∀*S* ∈ *H*, *x*, *y* ∈ *S*, *R*(*x*, *y*) ≥ *C*(*S*)(*x*). Moreover, the fuzzy strict preference relation *P* and the fuzzy indifference relation *I* corresponding to *R* are defined as follows: ∀*x*, *y* ∈ *X*, *P*(*x*, *y*) = max⁡{0, *R*(*x*, *y*) − *R*(*y*, *x*)}; *I*(*x*, *y*) = min⁡{*R*(*x*, *y*), *R*(*y*, *x*)}.

## 3. Fuzzy Sets *G*(*S*, *R*) and *M*(*S*, *R*)

We consider the following two crisp sets:

(1) $$\begin{array}{c} G_{c}\left(S,R_{c}\right)=\left\{x\in S\;|\;\forall y\in S,xR_{c}y\right\},\\ M_{c}\left(S,R_{c}\right)=\left\{x\in S\;|\;\forall y\in S,y{\overset{-}{P}}_{c}x\right\}, \end{array}$$

where *R*_*c*_ denotes the weak order (*⪰*) in the crisp case and *P*_*c*_ is the asymmetrical part of *R*_*c*_. In the crisp case, there exist certain connections between *G*_*c*_(*S*, *R*) and *M*_*c*_(*S*, *R*) [14]. In fuzzy environment, the membership function for the fuzzy set *G*(*S*, *R*) is *G*(*S*, *R*)(*x*) = *Min*⁡_∀*y*∈*S*_ *R*(*x*, *y*) [8]. Moreover, the membership function for the fuzzy set *M*(*S*, *R*) is easily obtained as follows:

(2) $$\begin{array}{c} M\left(S,R\right)\left(x\right)=1-\underset{\forall y\in S}{\mathrm{Max}}\left(R\left(y,x\right)-R\left(x,y\right)\right). \end{array}$$

**Lemma 6 (see [10])**

Let *R* be a fuzzy preference relation on *X* and ∀*S* ∈ *H*:

- *G*(*S*, *R*)⊆*M*(*S*, *R*);
- when *R* is strongly complete, *G*(*S*, *R*) = *M*(*S*, *R*).

**Remark 7**

Reference [10] discussed the relationships between the two fuzzy sets above in the more general framework.

In the crisp case, Sen [14] and Walker [15] discussed the nonemptiness of *G*_*c*_(*S*, *R*) and *M*_*c*_(*S*, *R*), respectively [14, 15]. Next, we discuss the conditions for the nonemptiness of the two fuzzy sets above.

**Proposition 8**

Let *R* be a fuzzy preference relation on *X* and ∀*S* ∈ *H*; if *R* is acyclic and complete, then *G*(*S*, *R*) is nonempty.

**Proposition 9**

Let *R* be a fuzzy preference relation on *X* and ∀*S* ∈ *H*; if *R* is acyclic, then *M*(*S*, *R*) is nonempty.

**Remark 10**

Propositions 8 and 9 provide a sufficient condition for the nonemptiness of the fuzzy sets *G*(*S*, *R*) and *M*(*S*, *R*), respectively. Moreover, Martinetti et al. (2014) investigated the conditions for the nonemptiness of them in the framework of the Georgescu choice function [16]. Therefore, the proofs of Propositions 8 and 9 are omitted.

Propositions 8 and 9 only give sufficient conditions for the nonemptiness of the fuzzy sets *G*(*S*, *R*) and *M*(*S*, *R*); the converses of them do not hold. Regarding their converses, Examples 11 and 12 are adequate.

**Example 11**

Let *X* = {*x*, *y*, *z*} and let *R* be a fuzzy preference relation on *X*. *R* is defined as follows:

(3) Rxyzx100.7y0.910z0.60.81

From *G*(*S*, *R*)(*x*) = min⁡_∀*y*∈*S*_ *R*(*x*, *y*), when *S* = {*x*, *y*, *z*}, *G*(*S*, *R*)(*z*) = 0.6 ≠ 0, so *G*(*S*, *R*) is nonempty. When *R*(*z*, *y*) > *R*(*y*, *z*) and *R*(*y*, *x*) > *R*(*x*, *y*) hold, *R*(*z*, *x*) = 0.6 < *R*(*x*, *z*) = 0.7. And hence *R* is not acyclic. Moreover, *R*(*x*, *y*) + *R*(*y*, *x*) = 0.9 < 1, *R*(*y*, *z*) + *R*(*z*, *y*) = 0.8 < 1, and hence *R* is not complete.

**Example 12**

Let *X* = {*x*, *y*, *z*} and let *R* be a fuzzy preference relation on *X*. *R* is defined as follows:

(4) Rxyzx10.80.6y0.710.5z0.70.31.

From *M*(*S*, *R*)(*x*) = 1 − *Max*⁡_*y*∈*S*_(*R*(*y*, *x*) − *R*(*x*, *y*)), when *S* = {*x*, *y*, *z*},  *M*(*S*, *R*)(*x*)  =  *M*(*S*, *R*)(*y*)  =  0.9,  *M*(*S*, *R*)(*z*) = 0.8, and hence *M*(*S*, *R*) is nonempty. When *R*(*x*, *y*) = 0.8 > *R*(*y*, *x*) = 0.7, *R*(*y*, *z*) = 0.5 > *R*(*z*, *y*) = 0.3, but *R*(*x*, *z*) = 0.6 < *R*(*z*, *x*) = 0.7, contradicting the acyclicity of *R*.

## 4. The Rationality of Fuzzy Choice Functions

### 4.1. Rationality Conditions of Fuzzy Choice Functions

Similar to the crisp case, some important rationality conditions of fuzzy choice functions were presented [10, 13], such as Weak Axiom of Fuzzy Revealed Preference (WAFRP), Strong Axiom of Fuzzy Revealed Preference (SAFRP), and Weak Fuzzy Congruence Axiom (WFCA). Next, parts of important rationality conditions are recalled.

**Condition 1**

For all *S*_1_, *S*_2_ ∈ *H*,  ∀*x* ∈ *S*_1_⊆*S*_2_, then *C*(*S*_2_)(*x*) ≤ *C*(*S*_1_)(*x*).

**Condition 2**

For all  *S*_1_, *S*_2_ ∈ *H*,  ∀*x*, *y* ∈ *S*_1_⊆*S*_2_, then

(5) $$\begin{array}{c} T\left(C\left(S_{1}\right)\left(x\right),C\left(S_{1}\right)\left(y\right),C\left(S_{2}\right)\left(x\right)\right)\leq C\left(S_{2}\right)\left(y\right). \end{array}$$

**Condition 3**

For all  *S*_1_, *S*_2_ ∈ *H*,  ∀*x*, *y* ∈ *S*_1_⊆*S*_2_, then

(6) $$\begin{array}{c} T\left(C\left(S_{1}\right)\left(x\right),C\left(S_{2}\right)\left(y\right)\right)\leq C\left(S_{2}\right)\left(x\right). \end{array}$$

**Condition 4**

For all  *S*_1_, *S*_2_ ∈ *H*, ∀*x*, *y* ∈ *S*_1_∩*S*_2_, *T*(*C*(*S*_1_)(*x*), *C*(*S*_1_)(*y*), *C*(*S*_2_)(*x*)) ≤ *C*(*S*_2_)(*y*).

*Weak Fuzzy Congruence Axiom (WFCA).* For all *S* ∈ *H*, ∀*x*, *y* ∈ *S*, *T*(*R*(*x*, *y*), *C*(*S*)(*y*)) ≤ *C*(*S*)(*x*).

**Remark 13**

Conditions 1–4 are fuzzy versions of several contraction-expansion conditions; WFCA is a version of WCA. For example, Condition 1 implies that the extent to which an alternative (*x*) is chosen in a set of alternatives (*S*_2_) is not larger than in the relevant subset (*S*_1_). In [13], Conditions 1–4 and WFCA of fuzzy choice functions and their relationships were investigated. In order to study further the properties of a fuzzy choice function, the following Assumption 14 is proposed.

**Assumption 14**

For all *S*_1_, *S*_2_ ∈ *H*, ∀*x* ∈ *S*_1_∩*S*_2_, then *C*(*S*_1_)(*x*) = *C*(*S*_2_)(*x*).

**Remark 15**

Assumption 14 implies that if an alternative* x *is included in two sets, then the extent to which* x* is chosen in those two sets is identical.

**Proposition 16**

Let *C* be a fuzzy choice function; if *C* satisfies Assumption 14, then it satisfies Conditions 2–4.

**Proof**

Let ∀*S*_1_, *S*_2_ ∈ *H*, ∀*x*, *y* ∈ *S*_1_∩*S*_2_,

(7) $$\begin{array}{c} T\left(C\left(S_{1}\right)\left(x\right),C\left(S_{2}\right)\left(y\right)\right)\leq T\left(1,C\left(S_{2}\right)\left(y\right)\right)=C\left(S_{2}\right)\left(y\right). \end{array}$$

Assumption 14 implies that if *S*_1_∩*S*_2_ = *S*_1_ and ∀*x* ∈ *S*_1_⊆*S*_2_, then *C*(*S*_1_)(*x*) = *C*(*S*_2_)(*x*). And hence, we have *T*(*C*(*S*_1_)(*x*), *C*(*S*_2_)(*y*)) ≤ *C*(*S*_2_)(*x*) since *C*(*S*) satisfies Assumption 14 and thus *C*(*S*) satisfies Condition 3. Consider

(8) T(C(S1)(x),C(S1)(y),C(S2)(x))  =T(T(C(S1)(x),C(S2)(x)),C(S1)(y))  ≤T(T(C(S1)(x),1),C(S1)(y))  =T(C(S1)(x),C(S1)(y))  ≤T(1,C(S1)(y))=C(S1)(y).

We have *C*(*S*_1_)(*y*) = *C*(*S*_2_)(*y*) since *C*(*S*) satisfies Assumption 14. And therefore, *T*(*C*(*S*_1_)(*x*), *C*(*S*_1_)(*y*), *C*(*S*_2_)(*x*)) ≤ *C*(*S*_2_)(*y*); thus *C*(*S*) satisfies Condition 4.

So *C*(*S*) also satisfies Condition 2 since Condition 4 implies Condition 2.

**Remark 17**

Proposition 16 indicates that a fuzzy choice function can satisfy three expansion conditions meanwhile in a certain assumption.

### 4.2. Rationality and Bounded Rationality of Fuzzy Choice Functions

**Definition 18**

A fuzzy choice function *C* is called rational if there exists a fuzzy relation *Q* such that ∀*S* ∈ *H*, ∀*x* ∈ *S*, *C*(*S*)(*x*) = *G*(*S*, *Q*)(*x*).

Moreover, if *R* is the fuzzy revealed preference associated with *C* and ∀*S* ∈ *H*, ∀*x* ∈ *S*, *C*(*S*)(*x*) = *G*(*S*, *R*)(*x*), then *C* is called normal [8].

**Definition 19**

A fuzzy choice function *C* is defined to be complete rational if and only if *C* is normal and the fuzzy revealed preference relation *R* is reflexive, complete, and* T*-transitive.

**Remark 20**

Complete rationality implies that the fuzzy choice of a decision maker is of a high degree of rationality. Moreover, the definition of complete rationality is different from Banerjee's [8]. The difference is that the characterization of transitivity in [8] is different from* T*-transitivity shown here. The transitivity in [8] needs to satisfy the following two conditions meanwhile: (1) ∀*x*, *y*, *z* ∈ *X*, min⁡(*R*(*x*, *y*), *R*(*y*, *z*)) ≤ *R*(*x*, *z*); (2) ∀*x*, *y*, *z* ∈ *X*; if *R*(*x*, *y*) ≥ *R*(*y*, *x*) and *R*(*y*, *z*) ≥ *R*(*z*, *y*), then *R*(*x*, *z*) ≥ *R*(*z*, *x*).

**Example 21**

Let *X* = {*x*, *y*, *z*}. A fuzzy choice function *C* is defined as follows:

(9) $$\begin{array}{c} C\left(\left\{x\right\}\right)\left(x\right)=C\left(\left\{y\right\}\right)\left(y\right)=C\left(\left\{z\right\}\right)\left(z\right)=1,\\ C\left(\left\{x,y\right\}\right)\left(x\right)=0.9,\;\;C\left(\left\{x,y\right\}\right)\left(y\right)=0.7,\\ C\left(\left\{x,z\right\}\right)\left(x\right)=0.8,\;\;C\left(\left\{x,z\right\}\right)\left(z\right)=0.5,\\ C\left(\left\{y,z\right\}\right)\left(y\right)=0.6,\;\;C\left(\left\{y,z\right\}\right)\left(z\right)=0.5,\\ C\left(\left\{x,y,z\right\}\right)\left(x\right)=0.8,\;\;C\left(\left\{x,y,z\right\}\right)\left(y\right)=0.6,\\ C\left(\left\{x,y,z\right\}\right)\left(z\right)=0.5. \end{array}$$

It is easy to check that *C* is complete rational.

**Definition 22**

A fuzzy choice function *C* is defined to be bounded rational if and only if there exists a reflexive, incomplete fuzzy relation *R* such that ∀*S* ∈ *H*, *C*(*S*) = *M*(*S*, *R*).

**Remark 23**

The incomplete fuzzy relation *R* on *X* refers to *R*(*x*, *y*) + *R*(*y*, *x*) < 1 for some *x*, *y* ∈ *X*. Bounded rationality implies that the fuzzy choice of a decision maker is of a certain degree of rationality, and its level of rationality is weaker than complete rationality.

**Example 24**

Let *X* = {*x*, *y*, *z*}. A fuzzy choice function *C* is defined as follows:

(10) $$\begin{array}{c} C\left(\left\{x\right\}\right)\left(x\right)=C\left(\left\{y\right\}\right)\left(y\right)=C\left(\left\{z\right\}\right)\left(z\right)=1,\\ C\left(\left\{x,y\right\}\right)\left(x\right)=0.6,\;\;C\left(\left\{x,y\right\}\right)\left(y\right)=1,\\ C\left(\left\{x,z\right\}\right)\left(x\right)=1,\;\;C\left(\left\{x,z\right\}\right)\left(z\right)=0.8,\\ C\left(\left\{y,z\right\}\right)\left(y\right)=0.4,\;\;C\left(\left\{y,z\right\}\right)\left(z\right)=1,\\ C\left(\left\{x,y,z\right\}\right)\left(x\right)=0.6,\;\;C\left(\left\{x,y,z\right\}\right)\left(y\right)=0.4,\\ C\left(\left\{x,y,z\right\}\right)\left(z\right)=0.8. \end{array}$$

Consider the fuzzy preference relation defined on *X* as follows:

(11) Rxyzx10.20.5y0.610.1z0.30.71

It is easy to check that *C* is bounded rational, and its level of rationality is weaker than complete rationality. For example, the extent to which *y* is chosen in the set {*x*, *y*} is higher than *x*, but the extent to which *x* is chosen in the set {*x*, *y*, *z*} is higher than *y*. This implies that the fuzzy preferences between alternatives *x* and *y* of a decision maker are inconsistent in different sets of alternatives. In other words, the fuzzy choice of the decision maker is of a certain degree of rationality.

**Definition 25 (see [8])**

Let *R* be a fuzzy preference relation on *X*. Let *S* ∈ *H* and *x* ∈ *S*. *x* is said to be relation dominant in *S* in terms of *R* if and only if *R*(*x*, *y*) ≥ *R*(*y*, *x*), ∀*y* ∈ *S*.

Wu et al. [13] proved that Condition 1 is a necessary condition; Conditions 2 and 3 and WFCA are, respectively, sufficient and necessary conditions of fuzzy choice function with complete rationality through fuzzy revealed preferences under the assumption that every involved choice set is normal (i.e., ∀*S* ∈ *H*, ∃*x* ∈ *S*, *C*(*S*)(*x*) = 1). Different from them, we study the rationality properties of the fuzzy choice function with bounded rationality by fuzzy preferences without the assumption that every involved choice set is normal.

**Proposition 26**

Let *C* be a fuzzy choice function; (1) if *C* is bounded rational, then *C* satisfies Condition 1; (2) if *C* is bounded rational and *y* is relation dominant in *S*_2_ in terms of *R*, then *C* satisfies Condition 2; (3) if *C* is bounded rational and *x* is relation dominant in *S*_2_ in terms of *R*, then *C* satisfies Condition 3.

**Proof**

(1) Let ∀*S*_1_, *S*_2_ ∈ *H*, ∀*x* ∈ *S*_1_, *S*_1_⊆*S*_2_,

(12) C(S1)(x)=M(S1,R)(x)=1−Max⁡y∈S1(R(y,x)−R(x,y)),C(S2)(x)=M(S2,R)(x)=1−Max⁡y∈S2(R(y,x)−R(x,y));

then *C*(*S*_2_)(*x*) − *C*(*S*_1_)(*x*) = *Max*⁡_*y*∈*S*_1__(*R*(*y*, *x*) − *R*(*x*, *y*)) − *Max*⁡_*y*∈*S*_2__(*R*(*y*, *x*) − *R*(*x*, *y*)) ≤ 0; that is, *C*(*S*_2_)(*x*) ≤ *C*(*S*_1_)(*x*).

(2) Let ∀*S*_1_, *S*_2_ ∈ *H*, ∀*x*, *y* ∈ *S*_1_,

(13) T(C(S1)(x),C(S1)(y),C(S2)(x))  =T(T(C(S1)(x),C(S2)(x)),C(S1)(y))  ≤T(T(C(S1)(x),C(S2)(x)),1)  =T(C(S1)(x),C(S2)(x))  ≤T(1,C(S2)(x))=C(S2)(x).

Since *y* is relation dominant in *S*_2_ in terms of *R*, and hence *R*(*y*, *x*) ≥ *R*(*x*, *y*), ∀*x* ∈ *S*_2_. Therefore,

(14) C(S2)(x)=M(S2,R)(x)=1−Max⁡y∈S2(R(y,x)−R(x,y))≤1−Max⁡x∈S2(R(x,y)−R(y,x))=C(S2)(y).

That is, *T*(*C*(*S*_1_)(*x*), *C*(*S*_1_)(*y*), *C*(*S*_2_)(*x*)) ≤ *C*(*S*_2_)(*y*).

(3) Let ∀*S*_1_, *S*_2_ ∈ *H*, ∀*x*, *y* ∈ *S*_1_; then *T*(*C*(*S*_1_)(*x*), *C*(*S*_2_)(*y*)) ≤ *T*(1, *C*(*S*_2_)(*y*))) = *C*(*S*_2_)(*y*).

Since *x* is relation dominant in *S*_2_ in terms of *R*, and hence *R*(*x*, *y*) ≥ *R*(*y*, *x*), ∀*y* ∈ *S*_2_. Therefore,

(15) C(S2)(y)=1−Max⁡x∈S2(R(x,y)−R(y,x))≤1−Max⁡y∈S2(R(y,x)−R(x,y))=C(S2)(x).

That is, *T*(*C*(*S*_1_)(*x*), *C*(*S*_2_)(*y*)) ≤ *C*(*S*_2_)(*x*).

**Remark 27**

Proposition 26 indicates that the fuzzy choice function with bounded rationality also satisfies Condition 1; this is consistent with the conclusion under complete rationality on one hand. The fuzzy choice function with bounded rationality satisfies Conditions 2 and 3 combined with other conditions, but its converse does not hold. This is different from the conclusion under complete rationality on the other hand.  Example 29 is given to illustrate the converse of Proposition 26.

**Corollary 28**

If *C* is bounded rational, ∀*S*_1_, *S*_2_ ∈ *H*,  ∀*x* ∈ *S*_1_, then *C*(*S*_1_ ∪ *S*_2_)(*x*) ≤ *C*(*S*_1_)(*x*)∨*C*(*S*_2_)(*x*).

**Example 29**

Let *X* = {*x*, *y*, *z*}. A fuzzy choice function *C* is defined as follows:

(16) $$\begin{array}{c} C\left(\left\{x\right\}\right)\left(x\right)=1,\;\;C\left(\left\{y\right\}\right)\left(y\right)=1,\;\;C\left(\left\{z\right\}\right)\left(z\right)=1,\\ C\left(\left\{x,y\right\}\right)\left(x\right)=0.6,\;\;C\left(\left\{x,y\right\}\right)\left(y\right)=0.2,\\ C\left(\left\{y,z\right\}\right)\left(y\right)=0.7,\;\;C\left(\left\{y,z\right\}\right)\left(z\right)=0.2,\\ C\left(\left\{x,z\right\}\right)\left(x\right)=0.5,\;\;C\left(\left\{x,z\right\}\right)\left(z\right)=0.3,\\ C\left(\left\{x,y,z\right\}\right)\left(x\right)=0.4,\;\;C\left(\left\{x,y,z\right\}\right)\left(y\right)=0.2,\\ C\left(\left\{x,y,z\right\}\right)\left(z\right)=0.2. \end{array}$$

In this example,

(17) $$\begin{array}{c} R=\left(\begin{array}{ccc} 1 & 0.6 & 0.5\\ 0.2 & 1 & 0.7\\ 0.3 & 0.2 & 1 \end{array}\right). \end{array}$$

It is easily checked that *C* satisfies Condition 1 and *R* is reflexive, incomplete, and acyclic. Since *M*({*x*, *y*}, *R*)(*x*) = 1 ≠ *C*({*x*, *y*})(*x*) = 0.6, *C*(*S*) ≠ *M*(*S*, *R*). Take *T* = min⁡; it is easily checked that *C* satisfies Conditions 2 and 3, but *C*(*S*) ≠ *M*(*S*, *R*).

**Proposition 30**

Let *C* be a fuzzy choice function; if *C* is bounded rational and *x* is relation dominant in *S* in terms of, then *C* satisfies WFCA.

**Proof**

(18) ∀S∈H,∀x,y∈S,T(R(x,y),C(S)(y))  ≤T(1,C(S)(y))=C(S)(y).

Since *x* is relation dominant in *S* in terms of *R*, *R*(*x*, *y*) ≥ *R*(*y*, *x*), ∀*y* ∈ *S*.

Therefore, *C*(*S*)(*y*) = 1 − *Max*⁡_*x*∈*S*_(*R*(*x*, *y*) − *R*(*y*, *x*)) ≤ 1 − *Max*⁡_*y*∈*S*_(*R*(*y*, *x*) − *R*(*x*, *y*)) = *C*(*S*)(*x*); that is, *T*(*R*(*x*, *y*), *C*(*S*)(*y*)) ≤ *C*(*S*)(*x*).

**Remark 31**

Proposition 30 indicates that the fuzzy choice function with bounded rationality also satisfies WFCA combined with other conditions; this is different from the conclusion under complete rationality.  Example 32 is given to illustrate the converse of Proposition 30.

**Example 32**

Let *X* = {*x*, *y*, *z*}. A fuzzy choice function *C* is defined as follows:

(19) $$\begin{array}{c} C\left(\left\{x\right\}\right)\left(x\right)=1,\;\;C\left(\left\{y\right\}\right)\left(y\right)=1,\;\;C\left(\left\{z\right\}\right)\left(z\right)=1,\\ C\left(\left\{x,y\right\}\right)\left(x\right)=0.6,\;\;C\left(\left\{x,y\right\}\right)\left(y\right)=0.2,\\ C\left(\left\{y,z\right\}\right)\left(y\right)=0.5,\;\;C\left(\left\{y,z\right\}\right)\left(z\right)=0.2,\\ C\left(\left\{x,z\right\}\right)\left(x\right)=0.6,\;\;C\left(\left\{x,z\right\}\right)\left(z\right)=0.3,\\ C\left(\left\{x,y,z\right\}\right)\left(x\right)=0.4,\;\;C\left(\left\{x,y,z\right\}\right)\left(y\right)=0.2,\\ C\left(\left\{x,y,z\right\}\right)\left(z\right)=0.2. \end{array}$$

In this example,

(20) $$\begin{array}{c} R=\left(\begin{array}{ccc} 1 & 0.6 & 0.6\\ 0.2 & 1 & 0.5\\ 0.3 & 0.2 & 1 \end{array}\right). \end{array}$$

Take *T* = min⁡; it is easily checked that *C* satisfies WFCA. Since *C*({*x*, *y*})(*x*) = 0.6 ≠ *M*({*x*, *y*}, *R*)(*x*) = 1, and hence *C*(*S*) ≠ *M*(*S*, *R*).

### 4.3. A Numerical Illustration

In this subsection, we will illustrate the theoretical analysis about bounded rationality with a simple example.

A city plans to build a hydroelectric plant; two experts are invited to give some opinions regarding the three technical alternatives* x*,* y,* and* z* for a better decision. Here experts' preferences and choices are fuzzy. The information collected from all experts is aggregated in the matrix *R* = (*R*_*ij*_)_*n*×*n*_; the number *R*_*ij*_ ∈ [0, 1],  *i*, *j* = 1,…, *n* shows the preference degree between alternatives.

The activity of one expert is materialized in the following preference matrices:

(21) $$\begin{array}{c} R=\left(\begin{array}{ccc} 1 & 0.6 & 0.2\\ 0.2 & 1 & 0.8\\ 0.6 & 0.2 & 1 \end{array}\right). \end{array}$$

For example the value *R*_23_ = 0.8 represents the degree to which the expert prefers alternative *y* to alternative *z*. Denote by *S* the subset of the set of alternatives {*x*, *y*, *z*}. The fuzzy choice function resulting from *R* is computed by

(22) $$\begin{array}{c} C\left(S\right)\left(x\right)=1-\underset{\forall y\in S}{\mathrm{Max}}\left(R\left(y,x\right)-R\left(x,y\right)\right). \end{array}$$

For example,

(23) C({x,y})(x)=1−Max⁡∀y∈{x,y}(R(y,x)−R(x,y))=1−Max⁡(0.2−0.6,0)=1.

After all computations, we obtain Table 1.

From the degree of the expert's inclinations, the extent to which *x* is chosen is less than *z* in the set of alternatives {*x*, *z*}, and therefore* z *will be chosen by the expert. However, the extent to which *z* is chosen is less than *x* in the set of alternatives {*x*, *y*, *z*}, and therefore *x* or *y* will be chosen by the expert. This implies that the expert's choices are inconsistent in different sets of alternatives including common alternatives. In other words, the fuzzy choices of the expert are irrational or bounded rational.

## 5. Conclusions

Many important rationality conditions have been put forward to study the rationality of fuzzy choice functions in different frameworks, and their relationships were also discussed. In this paper, two common fuzzy sets are studied and analyzed in the framework of the Banerjee choice function. Based on this, the concepts about complete rationality and bounded rationality of fuzzy choice functions are proposed, and some conclusions of this paper are as follows.

1. The nonemptiness for fuzzy sets *G*(*S*, *R*) and *M*(*S*, *R*) are studied and analyzed, respectively. Results show that the conclusion about the nonemptiness for the crisp set *M*_*c*_(*S*, *R*) can be extended to the corresponding fuzzy set, but not for the crisp set *G*_*c*_(*S*, *R*).
2. In a certain assumption, a fuzzy choice function can satisfy three expansion conditions meanwhile.
3. The complete rationality and bounded rationality of fuzzy choice functions are defined, respectively, based on *G*(*S*, *R*) and *M*(*S*, *R*). The bounded rationality of fuzzy choice function is studied, and results show that the fuzzy choice function with bounded rationality can satisfy some rationality conditions combined with a certain condition. Compared with complete rationality, the conclusions under bounded rationality are only necessary conditions.

The study on the rationality of fuzzy choice functions is a wide and deep research field. At present, the research on this subject is still in early stages, and it needs to be improved and perfected further. We only made a small part of the work, and the study on the bounded rationality of fuzzy choice functions still will be our research works in future.

## Figures and Tables

**Table 1:** The degree of the expert's inclinations.

The set of alternatives	{*xy*}	{*xz*}	{*yz*}	{*xy* *z*}
*C*(*S*)(*x*)	1	0.5	—	0.6
*C*(*S*)(*y*)	0.6	—	1	0.6
*C*(*S*)(*z*)	—	1	0.5	0.5
